# Phylogeny and differentiation of the St genome in *Elymus* L. sensu lato (Triticeae; Poaceae) based on one nuclear DNA and two chloroplast genes

**DOI:** 10.1186/s12870-015-0517-2

**Published:** 2015-07-12

**Authors:** Zhen-Zhen Dong, Xing Fan, Li-Na Sha, Yi Wang, Jian Zeng, Hou-Yang Kang, Hai-Qin Zhang, Xiao-Li Wang, Li Zhang, Chun-Bang Ding, Rui-Wu Yang, Yong-Hong Zhou

**Affiliations:** Triticeae Research Institute, Sichuan Agricultural University, Wenjiang 611130, Chengdu, Sichuan China; Key Laboratory of Genetic Resources and Crop Improvement, Ministry of Education, Sichuan Agricultural University, Wenjiang 611130, Chengdu, Sichuan China; College of Resources and Environment, Sichuan Agricultural University, Wenjiang 611130, Chengdu, Sichuan China; College of Life Science, Sichuan Agricultural University, Yaan, 625014 Sichuan China

**Keywords:** *Elymus* s. l., St genome, nr DNA, Chloroplast gene, Phylogeny, Molecular evolution

## Abstract

**Background:**

Hybridization and polyploidization can be major mechanisms for plant evolution and speciation. Thus, the process of polyploidization and evolutionary history of polyploids is of widespread interest. The species in *Elymus* L. sensu lato are allopolyploids that share a common St genome from *Pseudoroegneria* in different combinations with H, Y, P, and W genomes. But how the St genome evolved in the *Elymus* s. l. during the hybridization and polyploidization events remains unclear. We used nuclear and chloroplast DNA-based phylogenetic analyses to shed some light on this process.

**Results:**

The Maximum likelihood (ML) tree based on nuclear ribosomal internal transcribed spacer region (nrITS) data showed that the *Pseudoroegneria*, *Hordeum* and *Agropyron* species served as the St, H and P genome diploid ancestors, respectively, for the *Elymus* s. l. polyploids. The ML tree for the chloroplast genes (*mat*K and the intergenic region of *trn*H-*psb*A) suggests that the *Pseudoroegneria* served as the maternal donor of the St genome for *Elymus* s. l. Furthermore, it suggested that *Pseudoroegneria* species from Central Asia and Europe were more ancient than those from North America. The molecular evolution in the St genome appeared to be non-random following the polyploidy event with a departure from the equilibrium neutral model due to a genetic bottleneck caused by recent polyploidization.

**Conclusion:**

Our results suggest the ancient common maternal ancestral genome in *Elymus* s. l. is the St genome from *Pseudoroegneria*. The evolutionary differentiation of the St genome in *Elymus* s. l. after rise of this group may have multiple causes, including hybridization and polyploidization. They also suggest that *E. tangutorum* should be treated as *C. dahurica* var. *tangutorum*, and *E. breviaristatus* should be transferred into *Campeiostachys*. We hypothesized that the *Elymus* s. l. species origined in Central Asia and Europe, then spread to North America. Further study of intraspecific variation may help us evaluate our phylogenetic results in greater detail and with more certainty.

**Electronic supplementary material:**

The online version of this article (doi:10.1186/s12870-015-0517-2) contains supplementary material, which is available to authorized users.

## Background

Hybridization and polyploidization is a major mechanism in plant evolution and speciation [1, 2]. Polyploidization by itself has many consequences for genome evolution, particularly for gene expression and gene organization [3–5]. These changes may result in full fertility and stabilization of the hybrid condition and assist in establishing the phenotype in nature, which allows polyploids to adapt to new ecological niches or to be competitively superior to the parental donor [2, 6, 7].

Evolution under polyploidization alone and/or hybridization and polyploidization together can give rise to a complex of lineages whose phylogenetic relationships are unclear. For such groups, molecular genetic analysis is often necessary to elucidate the genome evolution patterns and the phylogenetic relationships among taxa [8].

The wheat tribe Triticeae (Poaceae) includes many different auto- and allopolyploid taxa, and has received considerable study of its systematics, genetics and speciation [9–11]. One example of a polyploid complex within that tribe Triticeae is the genus *Elymus* L. sensu lato delimited by Löve [12]; it is an important perennial genus with approximately 150 species worldwide. It includes the traditional species of *Elymus* L., *Roegneria* C. Koch, *Hystrix* Moench, *Sitanion* Raf., and *Kengyilia* C. Yen et J. L. Yang.

Since *Elymus* L. was first described as a genus by Linnaeus [13], its circumscription and taxonomy has changed through times but is still uncertain because of the huge morphological variation within and between species, the polyploid origin of the genus and the frequent spontaneous hybridizations between species [12, 14–16]. Löve [12] suggested that the taxonomic treatment for Triticeae species should be based on genomic constitution, recognizing StH to be the genomes of *Elymus*. Dewey [9] accepted Löve’s opinion but note the Y genome was represented in many Asiatic species, recommending that the genomic constitutions of *Elymus* should be StH, StY or StYH. *Roegneria* has been recognized a part of *Elymus* based on morphological characters: tufted plants; similar spikelets, one spikelet per node; lemma lanceolate-oblong, rounded ab-axially, 5-veined and veins connivent at apex; also they have a limited genomic relationship [10, 17, 18]. Although *Roegneria* shares one or more characteristics with *Agropyron*, *Elymus*, and *Kengyilia*, none have them in the same combination. Therefore, Baum *et al.* [19, 20] concluded that the genus *Roegneria* should be treated as a strictly separate from *Agropyron*, *Elymus*, and *Kengyilia*. The genus *Hystrix* was established by Moench with the *Hy. patula* as the type based on morphological character of lacking glumes, or possessing subulate or linear-setiform ones [21]. Dewey [9] and Löve [12] proposed to put *Hystrix* in *Elymus* based on the fact that *Hy. patula* contains the StH genome. However, it was suggested that species of *Hystrix* containing NsXm genomes, such as *Hystrix coreana*, *Hy. duthiei* ssp. *duthiei* and *Hy. duthiei* ssp. *longearistata* should be transferred into *Leymus* Hochst [22, 23]. The genus *Sitanion* Rafinesque was erected in 1819, and the type species was *Sitanion hystrix*. However, *Sitanion hystrix* and its varieties were treated as *Elymus hystrix* on the basis of cytogenetic studies [9, 24, 25]. The genus of *Kengyilia* C. Yen et J. L. Yang was described with *Kengyilia gobicola* C. Yen et J. L. Yang as the type species, which contains StYP genomes [26]. Based on the principle that taxonomic treatment should reflect phylogenetic history, Yen *et al.* [27] suggested that the genus *Elymus* s. l. should be split into *Elymus* sensu stricto (StH genome), *Roegneria* C. Koch (StY genome), *Australoroegneria* C. Yen & J. L. Yang (later renamed *Anthosachne* Steudel) (StYW genome), *Campeiostachys* Drob. (StYH genome), *Douglasdeweya* C. Yen, J. L. Yang & B. R. Baum (StP genome), and *Kengyilia* C. Yen et J. L. Yang (StYP genome) [28]. This change has been supported by a few taxonomists [8, 18, 27–32]. Also, some systematists have treated *Elymus* s. l. species as different genera, based on differences in morphology and the regional distribution of those polyploid species [27, 30, 32].

All *Elymus* s. l. species are allopolyploids ranging from tetraploids, hexaploids to octaploids [12]. Cytogenetic analyses suggested that the St-, H-, P-, W-genome originated from *Pseudoroegneria* (Nevski) Á. Löve, *Hordeum* L., *Agropyron* J. Gaertn., and *Australopyrum* (Tzvelev) Á. Löve, respectively [9, 12, 33]. No putative Y genome diploids have yet been identified [6, 9, 27, 33–35]. The St genome is the shared donor genome of the *Elymus* s. l. species which have StH, StY, StP, StYP, StYH and StYW genomes.

Several dioploid species with St genome in *Pseudoroegneria* occur from Ciscaucasica to the Middle East and Northern China, and on to western North America [12]. However, the evolutionary pathway of the St genome from dioploid *Pseudoroegneria* to *Elymus* s. l. via hybridization and polyploidization is still unclear.

Gaining a better understanding of the evolutionary history of polyploids is important to the study of plant evolution [1]. Molecular phylogenetic analyses have aided in this process [1, 36, 37]. Nuclear internal transcribed spacer (nrITS) DNA sequences have been used to study phylogenetic and genomic relationships at lower taxonomic levels [38–41]. The chloroplast DNA (cpDNA) sequences, including coding and non-coding regions such as *rbc*L gene, *mat*K gene, the intron of *trn*L and the intergenic spacer of *trn*L-*trn*F and *trn*H-*psb*A are also valuable source of markers for identifying the maternal donors of polyploids with additional capacity to reveal phylogenetic relationships of related species [38, 42–45]. In *Elymus* s. l., both nuclear and chloroplast genes have been used to identify genome donnors, to demonstrate hybridization events or introgression, to examine duplicate gene evolution, and to reveal the evolutionary history and origin of its species [38, 2, 3, 6–49].

In the present study, we analyzed the 6 accessions of 4 *Pseudoroegneria* species with St genome, 35 accessions of 12 other diploid species with P, W, V, H, I, E, Xp, Ns monogenome, and 28 *Elymus* s. l. allotetraploids using one internal transcribed spacer region of nuclear gene (nrDNA ITS) and two chloroplast genes (*mat*K and the intergenic region of *trn*H-*psb*A). The objectives of this study are: (1) to elucidate the phylogenetic relationships of some *Elymus* s. l. polyploid species; (2) to examine the genetic differentiation of St genome in *Pseudoroegneria*; (3) to investigate the genetic differentiation of St genome in polyploid *Elymus* s. l. relative to each other and *Pseudoroegneria*; (4) to compare the nucleotide diversity of the St-genome sequences of nrITS, *mat*K, and *trn*H-*psb*A between *Elymus* s. l. and its putative diploid donors and among *Elymus* s. l. species.

## Results

### Phylogeny analysis

#### nrITS analysis

**Fig. 1:**
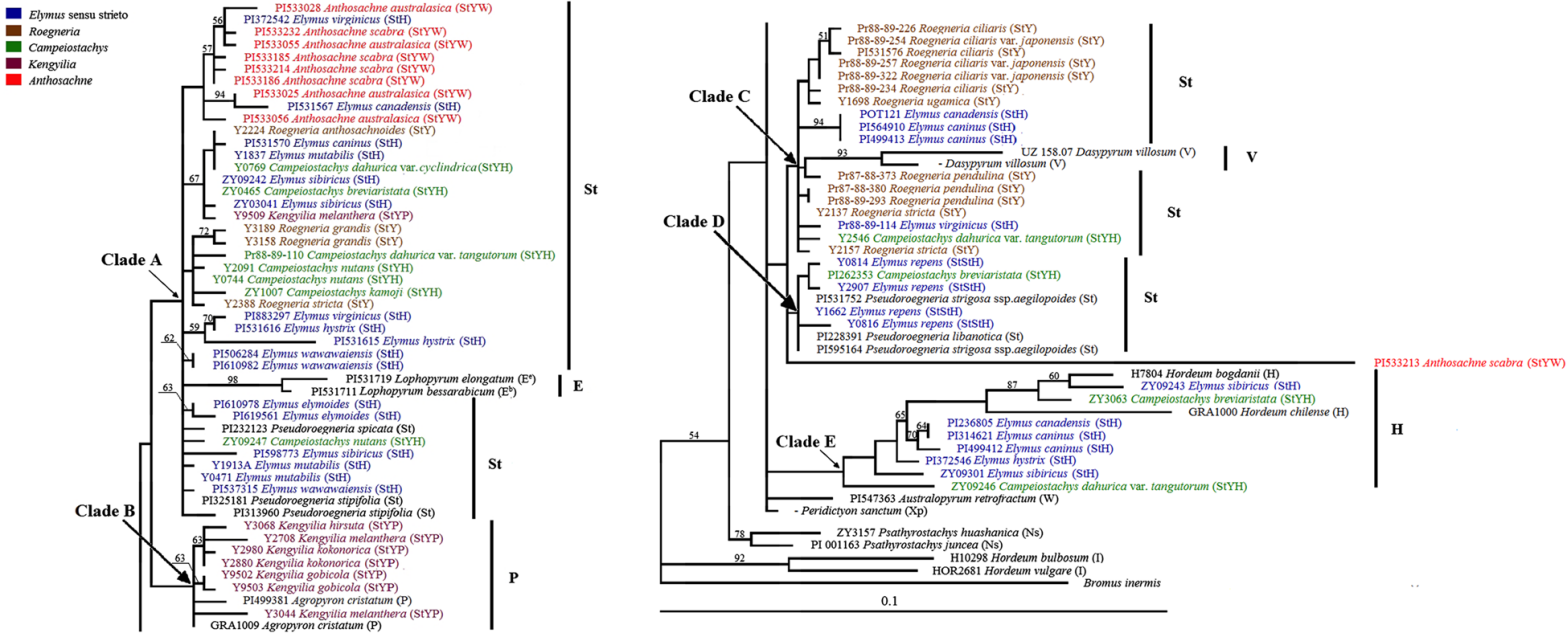
Maximum-likelihood tree (−Lnlikelihood = 3004.4870, base frequencies A: 0.21490, C: 0.26170, G: 0.27980, T: 0.24360, pinvar = none, shape = 0.5849) inferred from the nrITS sequences of *Elymus* L. sensu lato and its affinitive species, under GTR + G model. Numbers with bold above nodes are bootstrap values ≥ 50 %. The capital letters in bracket indicate the genome type of the species. Different color labeled the genera of *Elymus* L. sensu lato species

With the assumed nucleotide frequencies A: 0.21490, C: 0.26170, G: 0.27980, T: 0.24360, the nrITS data yielded a single phylogenetic tree (−Lnlikelihood = 3004.4870), the proportion of invariable sites = none, gamma shape parameter = 0.5849. Likelihood settings from best-fit model (GTR + G) selected by Akaike information criterion (AIC) in Modeltest 3.7. The ML tree with bootstrap support (BS) above branches was illustrated in Fig. 1. We obtained St-, P-, H-type nrITS sequences from our *Elymus* s. l. species.

The nrITS sequences from polyploidy were split into five clades (Clade A-E). Clade A included the St-genome sequences of *Pseudoroegneria* s*picata*, *Pse. stipifolia*, and nineteen *Elymus* s. l. species (*Anthosachne australasica*, *An. scabra* except PI533213; *Campeiostachys breviaristata* ZY0465, *C. dahurica* var. *clyindrica*, *C. dahurica* var. *tangutorum* Pr88-89 110, *C. kamoji*, *C. nutans*; *Kengyilia melanthera* Y9509; *Roegneria anthosachnoides*, *R. grandis*, *R. stricta* Y2388; *Elymus canadensis* PI531567, *E. caninus* PI531570, *E. elymoides*, *E. hystrix* except PI372546, *E. mutabilis*, *E. sibiricus* except ZY09243 and ZY09301, *E. virginicus* except Pr88-89-114, *E. wawawaiensis*) and the E^e^ genome sequence of *Lophopyrum elongatum* and E^b^ genome sequence of *L. bessarabicum*. Clade B consisted of the P-genome sequences of *Agropyron cristatum* and four *Kengyilia* species (*K. gobicola*, *K. kokonorica*, *K. melanthera* Y2078, Y3044 and *K. hirsuta* Y3068) species (76 % BS). In the Clade C, *Dasypyrum villosum* and nine *Elymus* s. l. species (*Roegneria ciliaris*, *R. ciliaris* var. *japonensis*, *R. ugamica*, *R. pendulina*, *R. stricta* Y2137 and Y2157; *Campeiostachys dahurica* var. *tangutorum* Y2546; *Elymus canadensis* POT121, *E. caninus* PI499413, PI564910 and *E. virginicus* Pr88-89-114) were gathered together. Two *Pseudoroegneria* species (*Pse. libanotica*, *Pse. strigosa* ssp. *aegilopoides*) and two *Elymus* s. l. species (*Campeiostachys breviaristata* PI262353; *Elymus repens*) formed Clade D. *Hordeum* species (*H. bogdanii* and *H. chilense*) and the H-genome sequences of six *Elymus* s. l. species (*Campeiostachys breviaristata* ZY3036, *C. dahurica* var. *tangutorum* ZY09246; *Elymus caninus* PI314621 and PI499412, *E. canadensis* PI236805, *E. hystrix* PI327546, *E. sibiricus* ZY09243 and ZY09301) comprised Clade E.

#### matK analysis

The ML analysis of the *mat*K sequence data yielded a single phylogenetic tree (−Lnlikelihood = 1787.3855), with the assumed nucleotide frequencies A: 0.36600; C: 0.15890; G: 0.17570; T: 0.29940, the proportion of invariable sites = none, gamma shape parameter = 0.8381. Likelihood settings from best-fit model (TVM + G) were selected by AIC in Modeltest 3.7. We found all *mat*k sequences from *Elymus* s. l. species corresponded to the St-type.

**Fig. 2:**
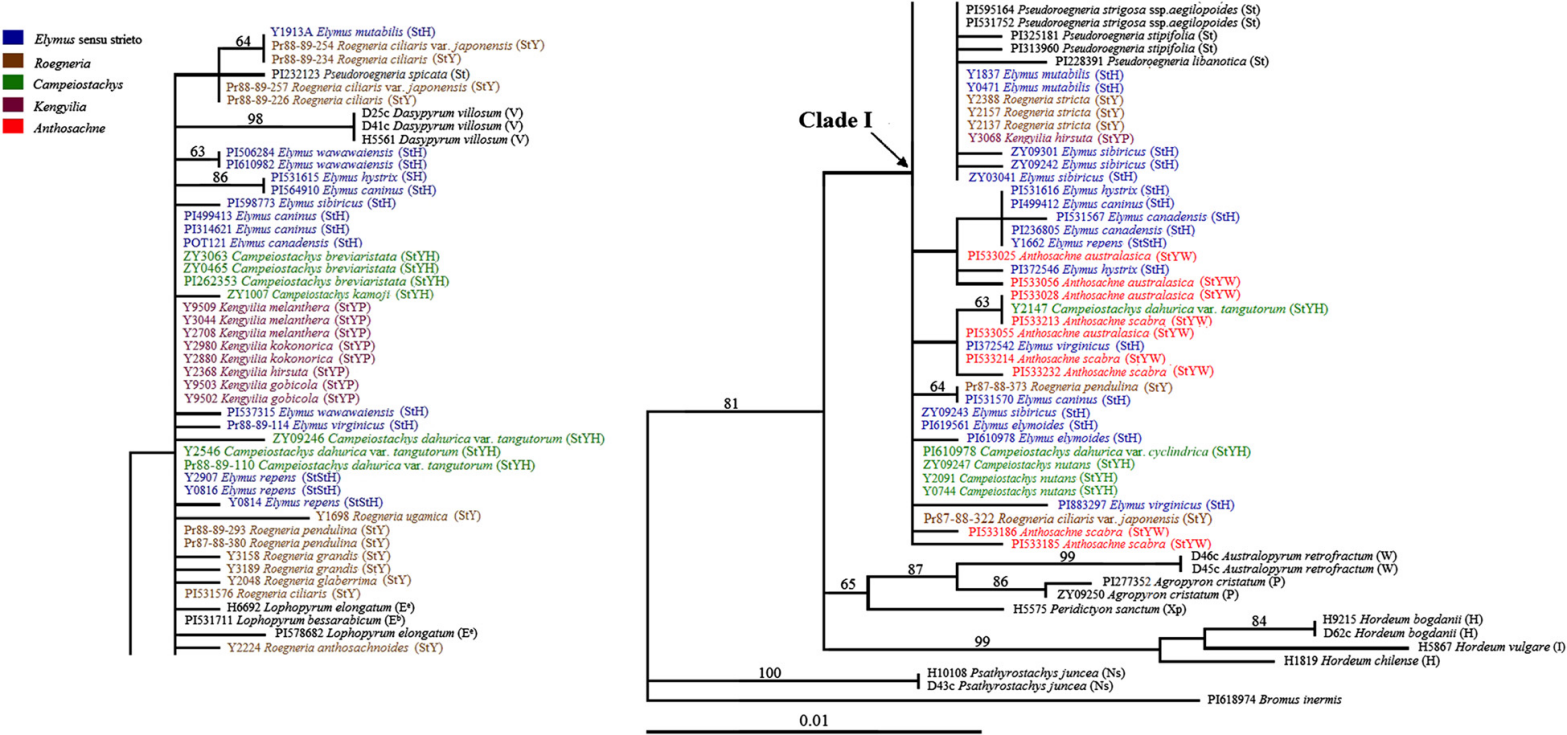
Maximum-likelihood tree (−Lnlikelihood = 1787.3855, base frequencies A: 0.36600; C: 0.15890; G: 0.17570; T: 0.29940, pinvar = none, shape = 0.8381) inferred from the *mat*K sequences of *Elymus* L. sensu lato and its affinitive species, under TVM + G model. Numbers with bold above nodes are bootstrap values ≥ 50 %. The capital letters in bracket indicate the genome type of the species. Different color labeled the genera of *Elymus* L. sensu lato species

The tree illustrated in Fig. 2 was ML tree for the *mat*K data with BS above branches. All the *Elymus* s. l. species and some diploid species of the Triticeae formed Clade I. The other diploid species were put outside Clade I. Within Clade I, the St-genome sequences of the following formed one subclade: all *Pseudoroegneria* species, the E^e^ genome sequence of *Lophopyrum elongatum*, the E^b^ genome sequence of *L. bessarabicum*, the V genome sequence of *Dasypyrum villosum* and twenty-three *Elymus* s. l. species (*Campeiostachys breviaristata*, *C. dahurica* var. *tangutorum* except Y2147, *C. kamoji*; *Kengyilia gobicola*, *K. kokonorica*, *K. melanthera* except Y9059, *K. hirsuta*; *Roegneria anthosachnoides*, *R. ciliaris*, *R. ciliaris* var. *japonensis* except Pr87-88-322, *R. glaberrima*, *R. grandis*, *R. pendulina* except Pr87-88-373, *R. ugamica*, *R. stricta*; *Elymus repens* except Y1662, *E. canadensis* POT121, *E. caninus* except PI499412 and PI531570, *E. hystrix* PI531615, *E. mutabilis*, *E. sibiricus* except ZY09243, *E. virginicus* Pr88-89-114, *E. wawawaiensis*). The other fourteen *Elymus* s. l. species (*Anthosachne australasica*, *An. scabra*; *Campeiostachys dahurica* var. *clyindrica*, *C. dahurica* var. *tangutorum* Y2147, *C. nutans*; *Roegneria pendulina*, *R. ciliaris* var. *japonensis* Pr87-88-322; *Elymus canadensis* except POT121, *E. caninus* PI499412 and PI531570, *E. elymoides*, *E. hystrix* except PI531615, *E. repens* Y1662, *E. sibiricus* ZY09243, *E. virginicus* except Pr88-89-114) were placed outside the subclade and formed a paraphyletic grade with a number of zero-length branches in the Clade I.

#### trnH-psbA analysis

**Fig. 3:**
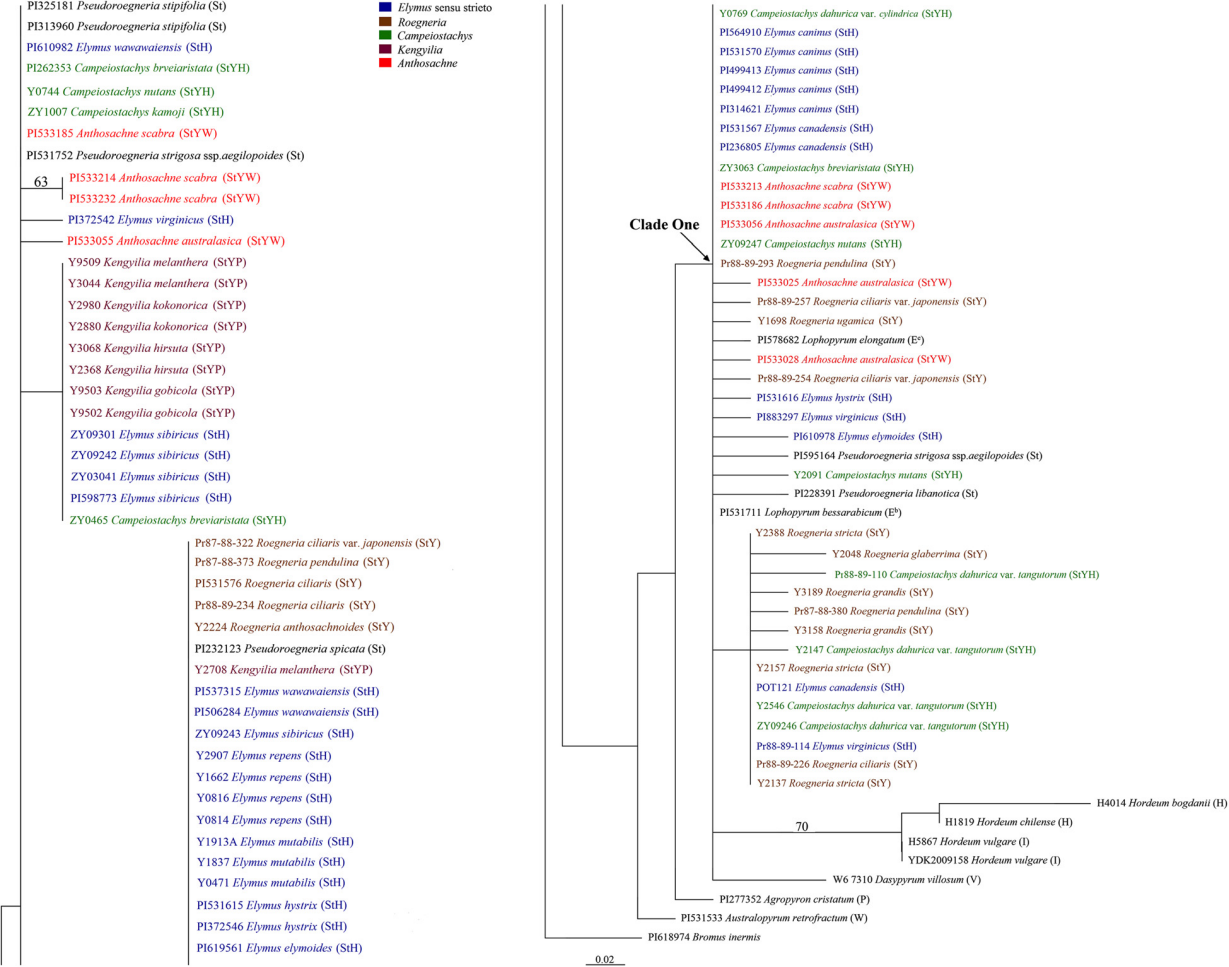
Maximum-likelihood tree (−Lnlikelihood = 1174.7281, base frequencies A: 0.35970; C: 0.17790; G: 0.18010; T: 0.28230, pinvar = none, shape = 0.1481) inferred from the *trn*H-*psb*A sequences of *Elymus* L. sensu lato and its affinitive species, under K81uf + G model. Numbers with bold above nodes are bootstrap values ≥ 50 %. The capital letters in bracket indicate the genome type of the species. Different color labeled the genera of *Elymus* L. sensu lato species

Likelihood settings from best-fit model (K81uf + G) were selected by AIC in Modeltest 3.7 (−Ln likelihood = 1174.7281). The assumed nucleotide frequencies A: 0.35970; C: 0.17790; G: 0.18010; T: 0.28230, the proportion of invariable sites = none, gamma shape parameter = 0.1481. The ML tree with BS above branches was illustrated in Fig. 3. We obtained two different St-type *trn*H-*psb*A sequences from *Elymus* s. l. species.

*Pseudoroegneria stipifolia*, *Pse. strigosa* ssp. *aegilopoides* PI531752 and twelve *Elymus* s. l. species (*Anthosachne*: *An. australasica* PI533055, *An. scabra* except PI533186 and PI533213; *Campeiostachys*: *C. breviaristata* except ZY3036, *C. kamoji* and *C. nutans* Y0744; *Kengyilia*: *K. gobicola*, *K. hirsuta*, *K. kokonorica*, *K. melanthera* except Y2708; *Elymus*: *E. wawawaensis* PI610982, *E. virginicus* PI372542 and *E. sibiricus* except ZY09243) were placed outside the Clade One, forming a paraphyletic grade with a number of zero-length branches in the ML tree inferred from the *trn*H-*psb*A data. Within Clade One, *Pse. libanotica*, *Pse. strigosa* ssp. *aegilopoides* PI595164, *Pse. spicata*, E^e^ genome sequence of *Lophopyrum elongatum* and the E^b^ genome sequence of *Lo. bessarabicum* were grouped with the other *Elymus* s. l. species. The H and I genome sequence of *Hordeum* species (*H. bogdanii*, *H. chilense, H. vulgare*) and the V genome sequence of *Dasypyrum villosum* were placed at the bottom of the Clade One.

### MJ-network analysis

As no recombination was detected using the GARD recombination-detection method within the HyPhy package, nrITS, *mat*K, and *trn*H-*psb*A sequences obtaitned in this study were used to generate MJ network. Each circular network node represents a single sequence haplotype, with node size being proportional to number of isolates with that haplotype. Median vectors (mv representing missing intermediates) show unsampled nodes inferred by MJ network analysis, and the number along the branches shows the position of mutations. Different species sharing a same haplotype circular network node were represented by distinct colors. Network loops represent either true reticulation events or alternative genealogies in closely related lineages.

**Fig. 4:**
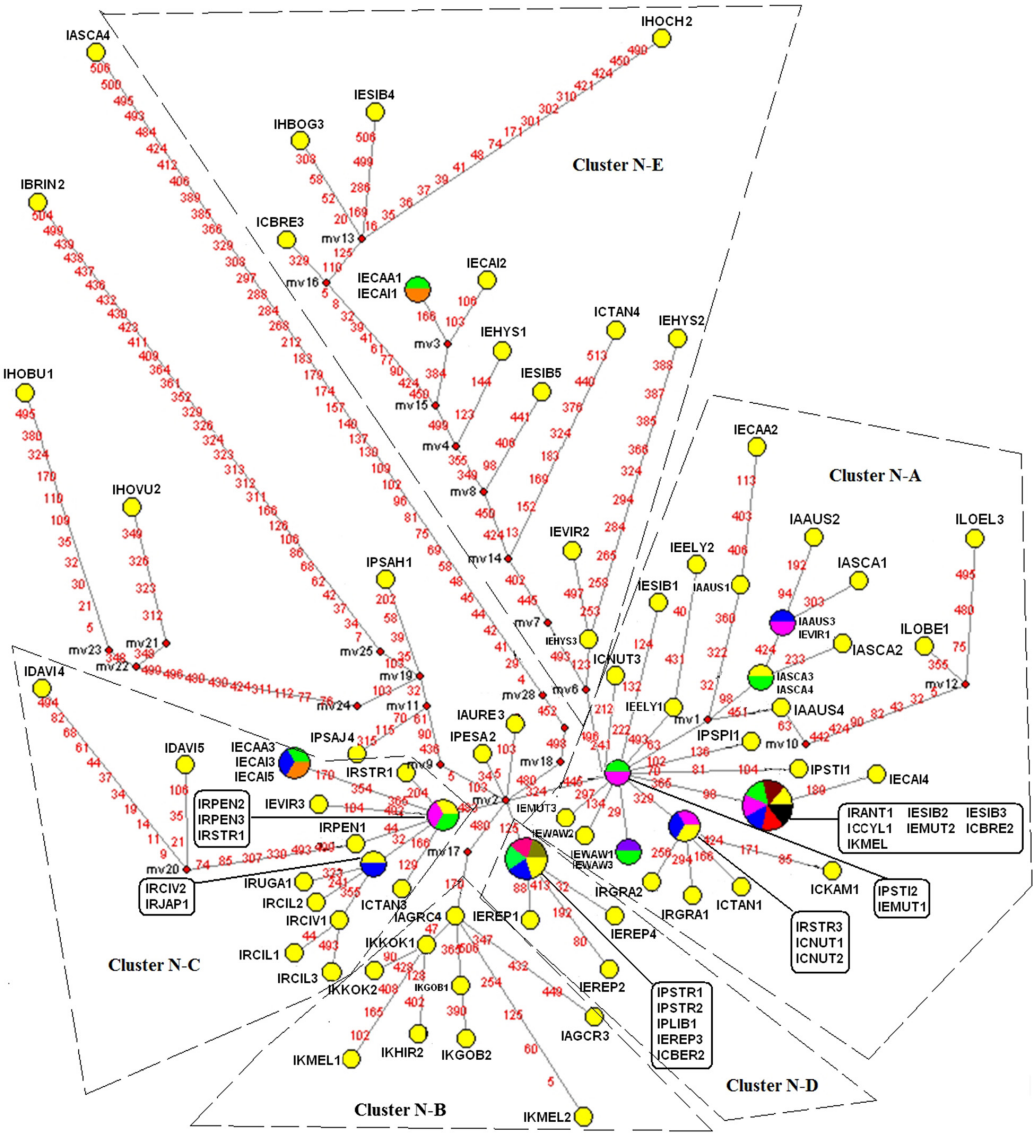
Median-joining networks based on nrITS locus haplotype of species of *Elymus* sensu lato, its dioploid donor and affinitive species. Haplotypes in network are represented by circles. Different species sharing a same haplotype circular network node were represented by distinct colors. Numbers along network branches indicate the position of mutation between nodes. Abbreviations of species names are listed in Additional file 1: Table S1. The numbers after species names represent different accessions of the same species

**Fig. 5:**
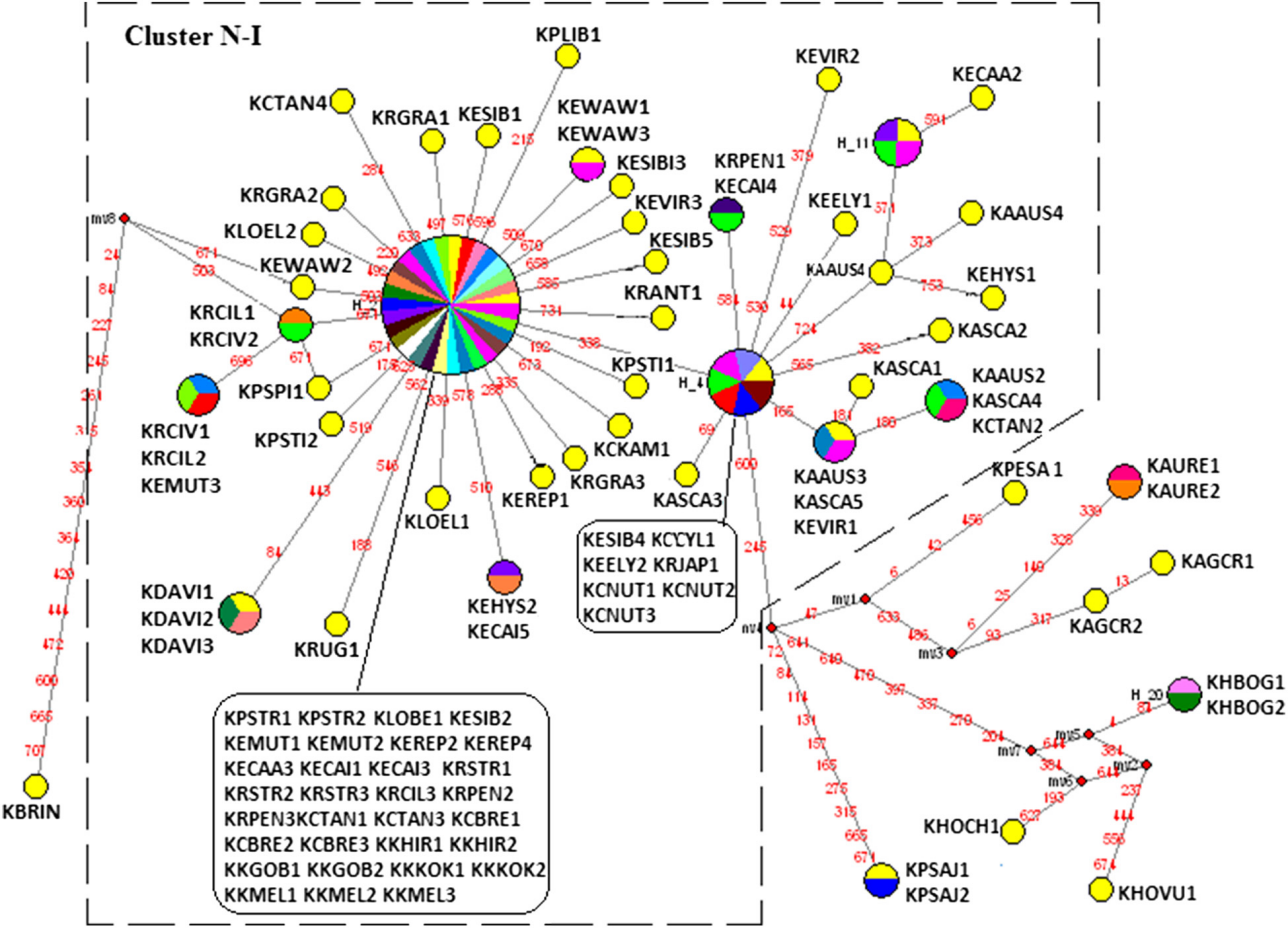
Median-joining networks based on *mat*K locus haplotype of species of *Elymus* sensu lato, its dioploid donor and affinitive species. Haplotypes in network are represented by circles. Different species sharing a same haplotype circular network node were represented by distinct colors. Numbers along network branches indicate the position of mutation between nodes. Abbreviations of species names are listed in Additional file 1: Table S1. The numbers after species names represent different accessions of the same species

**Fig. 6:**
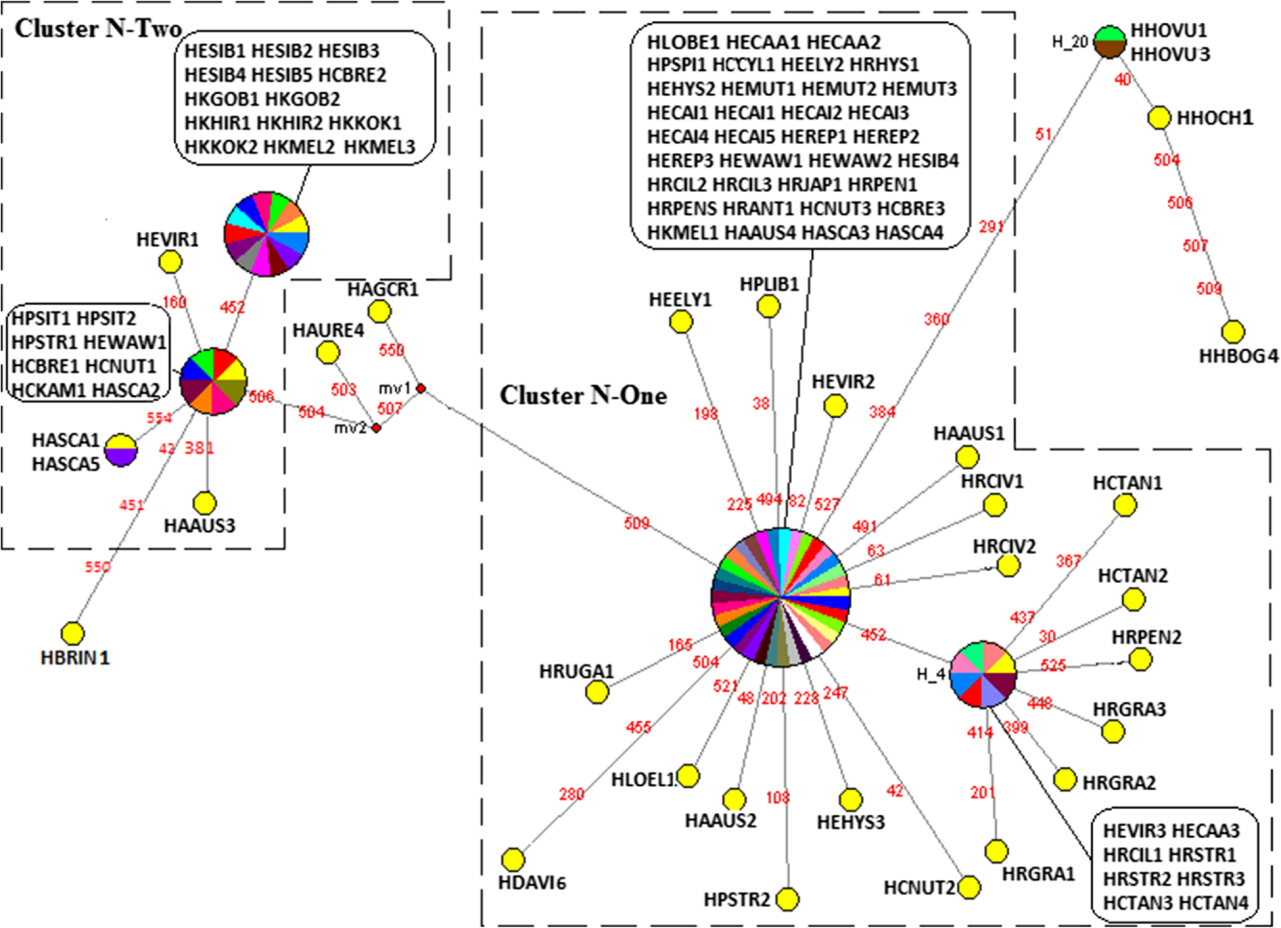
Median-joining networks based on *trn*H-*psb*A locus haplotype of species of *Elymus* sensu lato, its dioploid donor and affinitive species. Haplotypes in network are represented by circles. Different species sharing a same haplotype circular network node were represented by distinct colors. Numbers along network branches indicate the position of mutation between nodes. Abbreviations of species names are listed in Additional file 1: Table S1. The numbers after species names represent different accessions of the same species

Seventy-six, forty-eight, and thirty-three haplotypes were derived from 98 nrITS sequences (Fig. 4), 102 *mat*K sequences (Fig. 5), and 95 *trn*H-*psb*A sequences (Fig. 6), separately. We found median-joining (MJ) network showed a consistent phylogenetic reconstruction with ML tree. We identified those clusters’ name following the group name showed in the ML tree to make it clearly concerted. In the nrITS MJ network analysis, five clusters (Cluster N-A to Cluster N-E) representing three distinct types of haplotypes (St-, P-, and H-type) of *Elymus* s. l. In the *mat*K MJ-network analysis, all the species with St genome clustered together with St diploid species in Cluster N-I. The *trn*H-*psb*A MJ network analysis recognized two different St-types of haplotypes of *Elymus* s. l. species, grouped in Cluster N-One and N-Two.

### Nucleotide diversity analysis in St genome

Two measures of nucleotide diversity *π* and θ*w*, were separately calculated for each set of sequence data for the St genome of the diploid species (*Pseudoroegneria*), tetraploid StH and StY species and hexaploid StYW, StYH, StYP and StStH species. The Tajima’s test and Fu and Li’s test were conducted on each of different genome composing data sets (Table 1).

**Table 1:** Estimates of nucleotide diversity and test statistics at nrITS, matK and trnH-psbA locus on St-genome in species of Elymus sensu lato

	Gene	n	s	π	θ*w*	Fu & Li’s *D*	Tajima’s *D*
StH species	nrITS	512	46	0.0142	0.0250	−2.2572 (0.10 > *P* > 0.05)	−1.7192 (0.10 > *P* > 0.05)
	*mat*K	750	20	0.0035	0.0069	−2.5829 (*P* < 0.05)	−1.7801 (0.10 > *P* > 0.05)
	*trn*H-*psb*A	559	10	0.0039	0.0046	−1.2038 (*P* > 0.10)	−0.5366 (*P* > 0.10)
StY species	nrITS	499	22	0.0108	0.0133	−0.8768 (*P* > 0.10)	−0.7672 (*P* > 0.10)
	*mat*K	751	11	0.0024	0.0043	−1.8900 (*P* > 0.10)	−1.6981 (0.10 > *P* > 0.05)
	*trn*H-*psb*A	559	9	0.0026	0.0048	−2.5326 (*P* < 0.05)	−1.6134 (0.10 > *P* > 0.05)
StYW species	nrITS	516	14	0.0077	0.0105	−1.4635 (*P* > 0.10)	−1.3534 (*P* > 0.10)
	*mat*K	751	8	0.0032	0.0039	−0.7308 (*P* > 0.10)	−0.7695 (*P* > 0.10)
	*trn*H-*psb*A	565	9	0.0062	0.0059	−0.1073 (*P* > 0.10)	0.2621 (*P* > 0.10)
StYP species	nrITS	--	--	--	--	--	--
	*mat*K	754	4	0.0019	0.0020	−0.2642 (*P* > 0.10)	−0.0754 (*P* > 0.10)
	*trn*H-*psb*A	564	5	0.0020	0.0033	−1.8812 (0.10 > *P* > 0.05)	−1.6775 (0.10 > *P* > 0.05)
StYH species	nrITS	513	18	0.0104	0.0129	−0.7412 (*P* > 0.10)	−0.9514 (*P* > 0.10)
	*mat*K	750	6	0.0018	0.0027	−1.7133 (*P* > 0.10)	−1.1962 (*P* > 0.10)
	*trn*H-*psb*A	560	10	0.0059	0.0060	−0.5505 (*P* > 0.10)	−0.0104 (*P* > 0.10)
StStH species	nrITS	515	6	0.0062	0.0064	−0.3145 (*P* > 0.10)	−0.3145 (*P* > 0.10)
	*mat*K	754	4	0.0027	0.0029	−0.7801 (*P* > 0.10)	−0.7801 (*P* > 0.10)
	*trn*H-*psb*A	--	--	--	--	--	--
*Pseudoroegneria*	nrITS	514	9	0.0079	0.0077	−0.0922 (*P* > 0.10)	−0.1890 (*P* > 0.10)
	*mat*K	753	5	0.0022	0.0029	−1.3683 (*P* > 0.10)	−1.3370 (*P* > 0.10)
	*trn*H-*psb*A	564	8	0.0066	0.0062	0.0777 (*P* > 0.10)	0.3865 (*P* > 0.10)

The *n* is the number of the sites (excluding sites with gaps/missing data), *s* is the number of segregating sites, π is the average pairwise diversity, and θ_*w*_ is the diversity based on the number of segregating sites.

The St-type nrITS sequence of StYP species is missing from our data, thus we cannot report nucleotide diversity for that category. *trn*H-*psb*A sequences obtained from the StStH species (*Elymus repens*) were identical, in that case nucleotide diversity was zero. Tajima’s and Fu and Li’s D estimate for the *trn*H-*psb*A sequences from St genome species and Tajima’s D estimate for the *trn*H-*psb*A sequences from StYW genome species were positive, indicating a departure from the equilibrium neutral model at this locus, with an excess of rare sequence variants in the St genome diploid species and StYW genome hexaploid species based on *trn*H-*psb*A sequences.

## Discussion

### Phylogenetic relationships among the polyploids in ***Elymus*** s. l.

*Elymus* s. l. consists of allopolyploids that are widely distributed and includes a number of endemic species. Analyses of nrITS, *mat*K and *trn*H-*psb*A sequences collected from a wide range of *Elymus* s. l. species and related genera can shed light on their phylogenetic relationships, ancestral donors and the polyploidization events in the speciation processes on the basis of orthologous comparison.

The genus *Campeiostachys* was established using morphology based on a single species *Campeiostachys schrenkiana* (Fisch. & Mey. ex Schrenk) Drobov [50]. Baum *et al.* [32] suggested keeping the genus name *Campeiostachys* Drobov for the allohexaploid species with the genomic constitution StStHHYY which admittedly cannot be separated morphologically from the traditional genus *Elymus.* For example, based on the genome constitution, *Elymus breviaristatus* Keng was treated as *Campeiostachys breviaristata* (Keng) Y. H. Zhou, H. Q. Zhang et C. R. Yang. According to Yen *et al.* [51], *Elymus tangutorum* (Nevski) Hand. -Mazz was treated as *Campeiostachys dahurica* var. *tangutorum* in the *C. dahurica* complex [52]. Subtle morphological differences have often formed the basis for taxon recognition within the complex, resulting in different taxonomic treatments of the *Elymus dahurica* complex. The species complex possesses three haplomes St, Y, and H with 2n = 6x = 42 chromosomes and has an Asiatic distribution, ranging from Iran to Japan and from southern Siberia to central China [12]. Molecular diversity of the 5S rDNA units [53], storage proteins [54], and other considerations [55] in the *Elymus dahurica* complex supported the genomic constitution of St, Y, and H haplomes. The ML tree and MJ network based on nrITS data from this study, combined with unpublished GISH (Genomic in situ hybridization) results, confirms the genomic constitution of St, Y, and H haplomes in *E. tangutorum* and *E. breviaristatus*. Morphologically, *E. tangutorum* and *E. breviaristatus* are similar to the species in *Campeiostachys* in that they share the chatacteristic of palea and lemma having equal length [51]. Despite subtle morphological differences in these species, we strongly support the taxon treatment based on both genomic constitution and morphology. Thus, *E. tangutorum* should be treated as *C. dahurica* var. *tangutorum* and *E. breviaristatus* should be transferred into *Campeiostachys*.

It has been found recently that incomplete concerted evolution of nrDNA is widespread among angiosperms [56]. The frequency of heterogeneity among rDNA sequences is higher in alloployploids than that in diploid and autopolyploid species [57]. The main cause of heterogeneity is slowed concerted evolution due to hybridization and polyploidy. Concerted evolution in an allopolyploid may lead to a novel combination of nrITS sequences representing a mixture of the two original parental nrITS sequences that occur within a single individual. It is also possible that unidirectional concerted evolution could subsequently occur, leading to the loss of one copy and fixation of the new nrITS type. Furthermore, both types of parental sequences of the nrITS region could be maintained, especially in the case in young hybrid-derived taxa that have had little opportunity for concerted evolution [57–59]. In the present ML analysis, *Anthosachne scabra* (StYW, PI533213, Australia) was placed at abnormal branches site with St-type nrITS sequences obtained from *Pse. libanotica* (St, Middle East)*, Pse. strigosa* ssp. *aegilopoides* (St, PI595164, Central Asia; PI531752, Middle East), *Roegneria* (StY, Central Asia), *Campeiostachys* (StYH, Central Asia) and *Elymus* (StH, Central Asia and StStH, Central Asia). Additionally, a GA/GT insert at position 119–122 in the ITS sequence was detected for the *Roegneria* species in Clade C. At the same position, a GGT/AT insert in the nrITS sequence was detected for the *Elymus*, *Pseudoroegneria* and *Anthosachne scabra* (StYW, PI533213, Australia) species in Clade D. A CCAC insert at position 417–420 was detected for all species mentioned. And, these two clades were very close to each other. Thus, we hypothesized that the nrITS type obtained from this group might be a mid-type, representing a mixture of the two ancestral nrITS sequences (St-and St-Y-type). This situation may be due to inter-genome recombination, following hybridization either before or after the chromosome doubling event. Furthermore, *Pseudoroegneria* from Central Asia might have acted as an ancestor in the hybrid history of *Roegneria* (StY, Central Asia), resulting recombination sequences. Previous findings on the evolution of nrITS sequences in allopolyploids are typically similar to our findings; sequences that represent some combination of ancestral input [60, 61].

### The differentiation of St genome in ***Elymus*** s. l.

Prior research has demonstrated the evolutionary differentiation of the St genome in different diploid species. Considering the morphological differentiation of *Pseudoroegneria*, *Pse. stipifolia* has rough rachis densely covered by prickles; *P. spicata* has slender awns and unequal glumes; *Pse. strigosa* has long awns with equal glumes; but *Pse. tauri* and *Pse. libanotica* have no awns with unequal glumes [62]. The molecular data also shows differentiation in *Pseudoroegneria*. Sun *et al.* [63] reported a 39 bp MITE stowaway element insertion in the region of nuclear RNA polymerase II (RPB2) gene for *Pse. spicata* and *Pse. stipifolia*; *Pse. tauri* and *Pse. libanotica* lack this insertion. The *Pseudoroegneria* diploid species are widely distributed extending from Ciscaucasica to Middle East and Central Asia, and on to western of North America [12]. In our study, *Pse. libanotica* (Middle East)*, Pse. strigosa* ssp. *aegilopoides* (PI595164, Central Asia; PI531752, Middle East), *Pse. stipifolia* (Central Asia), and *Pse. spicata* (North America) were used in the phylogenetic analysis based on the nrITS, *mat*K and *trn*H-*psb*A data. All *Elymus* s. l. species grouped with the *Pseudoroegneria* species in the ML tree and MJ network using the *mat*K data. Although in the ML tree and MJ network based on the *trn*H-*psb*A data, *Pse. stipifolia* from Central Asia and *Pse. strigosa* ssp. *aegilopoides* (PI531752) from Middle East were closely placed with six *Elymus* s. l. tetraploids and sixteen *Elymus* s. l. hexaploids, *Pseudoroegneria libanotica* and *Pse. strigosa* ssp. *aegilopoides* (PI595164) from Middle East and Central Asia, *Pse. spicata* from North America were grouped with the rest *Elymus* s. l. species. Similar results were obtained in the ML tree and MJ network based on the nrITS sequence data. Collectively, the results implied that the *Pseudoroegneria* species from Central Asia and Middle East are more ancient than those from North America. Obviously, the differentiation exists in the diploid *Pseudoroegneria* species from Middle East, Central Asia and North America. The formation of *Pseudoroegneria* species appear to have originated in the Central Asia and Europe, later spreading to North America.

In this study, based on the *mat*K data, all the *Elymus* s. l. species were grouped with the *Pseudoroegneria* species (with sub-clades) in the ML tree and MJ network. In contrast, the ML tree and MJ network based on the *trn*H-*psb*A data closely placed *Pse. stipifolia* from Central Asia and *Pse. strigosa* ssp. *aegilopoides* (PI531752) from Middle East with three tetraploids (*E. wawawaensis*, *E. virginicus* and *E. sibiricus*) and nine hexaploids (*C. breviaristata, C. kamoji*, *C. nutans*, *An. australasica*, *An. scabra*, *K. gobicola*, *K. hirsuta*, *K. kokonorica* and *K. melanthera*). *Pseudoroegneria libanotica* and *Pse. strigosa* ssp. *aegilopoides* (PI595164) from Middle East and Central Asia, *Pse. spicata* from North America were grouped with the rest *Elymus* s. l. species. Similar results were obtained from the ML tree based on the nrITS sequence data. The evolution of *Elymus* s. l. species might appears to parallel that of the *Pseudoroegneria* species, originating in Central Asia and Europe, then spreading to the North America via recurrent hybridization and polyploidization events. In addition, *Elymus* s. l. species were split into different St-groups. For instance, two accessions of hexaploid *C. breviaristata* were placed in separate St-genome clade in the ML tree based on the nrITS and *trn*H-*psb*A sequence data. The same situation was also detected in the tetraploid *E. canadensis* in the ML tree based on the *mat*K and *trn*H-*psb*A sequence data. Such patterns indicate that differentiation of St genome existed in the species of *Elymus* s. l. at both the genus and species after polyploidization event based on the nrDNA ITS and the chloroplast *mat*K and *trn*H-*psb*A molecular data. We also found non-coding cpDNA sequences (*trn*H-*psb*A) provided more phylogenetic information than coding cpDNA sequences (*mat*K), revealing the differentiation of St genome in *Elymus* s. l. species more clearly.

Evolutionary dynamics of duplicate genes can provide a better understanding of the processes of polyploidization and subsequent rapid diversification [1, 4]. In this study, nrITS and *mat*K nucleotide sequence diversity of the St genome of tetraploid StH and StY tetraploid species was higher than in the St genome of diploid *Pseudoroegneria*. Tajima’s and Fu and Li’s *D* estimate for the *trn*H-*psb*A in the St genome of diploid *Pseudoroegneria* was positive. This result indicated a departure from the equilibrium neutral model at this locus, with an excess of rare sequence variants in the diploid *Pseudoroegneria* species. This finding is compatible with a genetic bottleneck created by recent polyploidization during radiation of *Pseudoroegneria* species. The values of Tajima’s and Fu and Li’s *D* statistic for nrITS, *mat*K and *trn*H-*psb*A sequence on StH and StY genome were all negative*,* indicating that the observed number of rare variations exceeds the expected number in an equilibrium neutral model. These estimates indicated that the excess of rare variants in tetraploid StH and StY species might be created by different independent hybridization event or introgression of St genome during polyploidization.

Our phylogenetic results support the possibility that StY tetraploid species was the direct ancestor of the StYW, StYP and StYH hexaploid species during the allohexaploid speciation process (see next discussion section). We compared the nucleotide sequence diversity of the nrITS, *mat*K and *trn*H-*psb*A between the St genome of StY tetraploid spices and the StYW, StYP and StYH hexaploid spices, respectively. As the narrow distribution of StYW and StYP species and rare species of StYH species compared with StY species, the nucleotide sequence diversity in the St genome of tetraploid StY species were higher than in the St genome of hexaploid species (StYH and StYW for nrITS, *mat*K sequence, and StYP for *mat*K and *trn*H-*psb*A sequence). In addition, the values of Tajima’s and Fu and Li’s *D* statistic for nrITS, *mat*K and *trn*H-*psb*A gene loci of the St genome of hexaploid StYW, StYP and StYH species (except the Tajima’s *D* for *trn*H-*psb*A gene on the St genome of hexaploid StYW species) were negative, indicating that the observed number of rare variations exceeds the expected number in an equilibrium neutral model. These estimates indicated that the excess of rare variants in hexaploid StYH, StYW and StYP species also have been created by different independent hybridization or introgression events of St genome during polyploidization.

### Putative origins of the polyploids in ***Elymus*** s. l.

Cytogenetical studies have concluded that *Pseudoroegneria*, *Hordeum*, *Australopyrum*, and *Agropyron* species have served as the St, H, W, and P genome diploid donors, respectively, during the polyploid speciation of *Elymus* s. l. species [9, 17, 35]. In the ML tree based on the nrITS data, three types of nrITS sequences (St-, H- and P-type) were obtained from all the polyploidy *Elymus* s. l. species (except the *An. scabra* PI533213) in the present study. This result indicated that nrITS sequences in different *Elymus* s. l. species were very similar to their diploid ancestors, confirming that *Elymus* s. l. is closely related to *Pseudoroegneria*, *Hordeum* and *Agropyron*. Combined with the prior cytogenetic results, we can conclude that the *Pseudoroegneria*, *Hordeum* and *Agropyron* species served as the St, H and P genome diploid donors during the allopolyploid speciation of *Elymus* s. l. species. Our conclusion is partly consistent with prior the single-copy nuclear gene data (*Acc1* and *Pgk1*) studies [8]. Those studies also proposed that *Australopyrum* species served as the W genome diploid donors during the polyploid speciation of *Anthosachne* species. We did not obtain W-type nrITS sequences in this study. In a future study the W-type nrITS sequences from *Anthosachne* might be obtained by screening a larger number positive clones with the nrITS sequence insert to test whether *Australopyrum* contributed to the evolution of *Elymus* s. l. species.

Phylogenetic analysis of our nrITS data revealed each homoeologous sequence grouped with those from the corresponding diploid progenitors. Similarly, the homoeologous loci of nrITS from sampled StYH genome *Campeistachys* species (*C. komoji* and *C. nutans*), StYP genome *Kengyilia* species (*K. melanthera*) and StYW genome *Anthosachne* species (*An. scabra* and *An. australasica*) were recovered, with each homoeologous locus also grouping with the StY genome *Roegneria* species (*R. anthosachnoid*, *R. grandis* and *R. stricta*) and StH genome *Elymus* sensu stricto species (*E. canadensis*, *E. caninus*, *E. elymoides*, *E. hystrix*, *E. mutabilis*, *E. sibiricus*, *E. virginicus* and *E. wawawaiensis*). These results strongly support the suggestion that the StYH, StYP and StYW genome species had their allohexaploid origin via StY as one of the hybridizing ancestors. Combined with the previous cytogenetic evidence, relatively large population size of the StY genome *Roegneria* species and the failure to discover the diploid Y-genome donor, it can be concluded that the StY genome species might serve as a direct donor of the StYH, StYP and StYW genome species during the allohexaploid speciation. These results also suggested a multiple origin of some polyploid species resulting from independent origin. This conclusion is compatible with the hypothesis of Yen *et al.* [27] and the results of Fan *et al.* [8]

## Conclusion

In this study, the nrITS sequence analysis in different *Elymus* s. l. species showed a clear linkage between nrITS sequences of polyploid *Elymus* s. l. species and those of their diploid ancestors. Combined with the previous cytogenetic results, our data supported the premise that *Pseudoroegneria*, *Hordeum* and *Agropyron* species served as the St, H and P genome diploid donors during the polyploid speciation of *Elymus* s. l. species. Analyses of phylogenetic relationships based on nrITS data also showed that it is reasonable to treat the *E. tangutorum* as *C. dahurica* var. *tangutorum* and transfer the *E. breviaristatus* into *Campeiostachys* in spite of subtle morphological differences in these species. We strongly support the taxonomy according to both genomic constitution and morphology. Sequence diversity patterns analyses of the two chloroplast genes suggested that the *Pseudoroegneria* (St genome donor) served as the maternal donor during the polyploidization events that gave rise to *Elymus* s. l. Those patterns also suggested that *Pseudoroegneria* species from Central Asia and Europe were more ancient than those from North America. *Elymus* s. l. species appear to have originated in Central Asia and Europe, then spread to the America after the recurrent hybridization and polyploidization events. Furthermore, differentiation of St genome existed at both genus and species level based on the nrDNA ITS and the chloroplast *mat*K and *trn*H-*psb*A sequences. The molecular diversity of the two chloroplastid genes and one nuclear DNA sequence in the St genome reflect the evolution of the St genome in the *Elymus* s. l. The molecular evolution in the St genome may go into a period of non-random evolution following the polyploidization event and introgression of St genome departing from the equilibrium neutral model due to a genetic bottleneck caused by recent polyploidization.

## Methods

### Taxon sampling

Twenty-eight *Elymus* s. l. species were included in this study and were analyzed together with sixteen diploid taxa representing nine basic genomes in the tribe Triticeae (See Additional file 1: Table S1). *Bromus inermis* Leyss was used as outgroup. The seed materials with PI numbers were kindly provided by American National Plant Germplasm System (Pullman, Washington, USA). We collected the seed materials with Pr, ZY, and Y numbers. The plants and voucher specimens were deposited at Herbarium of Triticeae Research Institute, Sichuan Agricultural University, China (SAUTI).

### DNA extraction, amplification and sequencing

The CTAB (Cetyltrimethyl Ammonium Bromide) procedure [64] was used to isolate total DNA. The nuclear nrITS sequence, chloroplast *mat*K and *trn*H-*psb*A spacer sequence were amplified with primers listed in Table 2. PCR amplification of the cpDNA was carried out in a 50 μL reaction mixture, containing 10× ExTaq polymerase buffer, 2 mM MgCl_2_, 200 μM of dNTP, 1 μM of each primer, 1.5 U ExTaq and about 30 ng of template DNA. Amplifications were performed on Mastercycler (Pro S, Eppendorf, Germany) using protocols described in Table 3. The PCR products were visualized on 1.0 % agarose gels, purified by an ENZA™ gel extraction kit (Omega Bio-Tech, Georgia, USA) and then cloned into pMD19-T vector (TaKaRa, Dalian, China) according to the manufacturer’s instructions. Three random clones per diploid were chosen to sequence. As there are at least three to five accessions for each allopolyploid in this study, only one random clone for each accession of allopolyploid was picked and sequenced. All clones were sequenced in both directions in Beijing Genomics Institute (BGI, Beijing, China).

**Table 2:** Names, sequences, and references of primers used in this study

Gene	Name of primers	Sequence of primer (5′-3′)	Reference
nrITS	ITS4	TCCTCCGCTTATTGATAGC	White *et al.* [71]
	ITS5	GGAAGTAAAAGTCGTAACAAGG	
*mat*K	W	TACCCTATCCTATCCAT	Hilu *et al.* [72]
	9R	GCTAGAACTTTAGCTCGTA	
*trn*H-*psb*A	trnH1	GTTATGCATGAACGTAATGCTC	Shaw *et al.*[73]
	trnH2	CGCGCATGGTGGATTCACAATCC

**Table 3:** Thermocycling conditions for amplification of genes using the PCR

Gene	Protocol
nrITS	1 cycle: 5 min 95 °C
	35 cycles: 1 min 94 °C, 1 min52 °C, 1 min 72 °C
	1 cycle: 8 min72 °C
*mat*K	1 cycle: 4 min 95 °C
	35 cycles: 1 min 94 °C, 1 min50 °C, 1.5 min 72 °C
	1 cycle: 10 min72 °C
*trn*H-*psb*A	1 cycle: 4 min 95 °C
	25 cycles: 1 min 94 °C, 1 min56 °C, 1 min 72 °C
	1 cycle: 7 min72 °C

### Phylogenetic analysis

Multiple sequences alignments were made using ClustalX [65], with additional manual adjustment. Phylogenetic analyses were performed using Maximum likelihood (ML). Maximum likelihood analyses of the nrITS data, *mat*K data and *trn*H-*psb*A data were performed in PAUP*4.0b10 (Swofford D L, Sinauer Associates, http://www.sinauer.com). The evolutionary model used for the phylogenetic analyses was determined using ModelTest v3.0 with Akaike information criterion (AIC) [66]. The optimal model were GTR + G for nrITS data, TVM + G for *mat*K data, and K81uf + G for *trn*H-*psb*A data. Maximum likelihood heuristic searches were performed with 100 random addition sequence replications and Tree Bisection-Reconnection (TBR) branch swapping algorithm. In order to infer the robustness of clades, bootstrap support (BS) values were calculated with 1000 replications [67].

### Network analysis

Taking into consideration the potential for reticulation in the evolution of polyploids, phylogenetic network reconstruction method was used to study the relationship between ancestral and derived haplotypes in this study. Because we used known gene genealogies in our simulation studies, the median-joining (MJ) network method was performed [68]. The MJ network method has already been successfully used to study the specific progenitor-descendant relationship of polyploidy Triticeae species [69, 70, 11]. The MJ network analysis was generated by the Network 4.6.1.3 program (Fluxus Technology Ltd, Clare, Suffolk, UK). Because the program infers median-joining networks from non-recombining DNA [71], the GARD recombination detection method within the HyPhy package [72] was used to test for recombination.

### Nucleotide diversity estimate

To assess the gene divergence and genetic relationships in the St genome between polyploids and its diploid progenitor, nucleotide diversity was estimated by Tajima’s π [73], and Watterson’s θ [74, 75]. Tajima’s π quantifies the mean percentage of nucleotide differences among all pairwise comparisons for a set of sequences, while Watterson’s θ is simply an index of the number of segregating (polymorphic) sites. Tests of neutrality including Tajima’s and Fu and Li’s *D* statistic were performed as described by Tajima [73], and Fu and Li [76]. Significance of *D*-values was estimated with the simulated distribution of random samples (1000 steps) using a coalescence algorithm assuming neutrality and population equilibrium [77]. These parameters were calculated with DnaSP 4.10.9 [78].

### Availability of supporting data

The data sets supporting the results of this article were deposited in the GenBank (http://www.ncbi.nlm.nih.gov) repository. The phylogenetic trees were deposited in treebase (http://treebase.org) under following URL: http://purl.org/phylo/treebase/phylows/study/TB2:S17529?x-access-code=6966b2e62a8abd50894460721ce2f4b7&format=html. The data sets supporting the results of this article are included within the article and its additional file.

## Additional file

Additional file 1: Table S1. Species of *Elymus* sensu lato and the related species used in this study.

## Abbreviations

ML: Maximum likelihood
nrITS: Nuclear ribosomal internal transcribed spacer
GISH: Genomic in situ hybridization
CTAB: Cetyltrimethyl ammonium bromide
AIC: Akaike information criterion
TBR: Tree bisection-reconnection
BS: Bootstrap support
MJ: Median-joining
